# Confusion With Presentations of Calcium Pyrophosphate Dihydrate Disease: A Report of Two Cases Mistaken for Cellulitis

**DOI:** 10.7759/cureus.34789

**Published:** 2023-02-08

**Authors:** Masooma S Rana, Mahanoor Raza, Mobeena Arif, Taofeek Akinpelu, Abdul Waheed

**Affiliations:** 1 Family Medicine, Aga Khan University Hospital, Karachi, PAK; 2 Family Medicine, WellSpan Good Samaritan Hospital, Lebanon, USA; 3 Family Medicine, Wellspan Good Samaritan Hospital, Lebanon, USA; 4 Family and Community Medicine, Penn State University College of Medicine, Milton S. Hershey Medical Center, Hershey, USA

**Keywords:** pseudogout, crystal arthropathy, pseudocellulitis, cellulitis, cppd arthritis, calcium pyrophosphate dihydrate crystal deposition

## Abstract

Both pseudogout and cellulitis are diseases that may mimic one another in clinical practice. We discuss two cases of acute calcium pyrophosphate dihydrate (CPPD) arthritis mistaken for cellulitis in the emergency department. Both patients experienced significant improvement after management was changed to treat CPPD. These cases highlight how it is essential for physicians to consider CPPD as a differential diagnosis for a patient that is presenting with signs of inflammation in any joint.

## Introduction

Pseudogout is a crystal-induced arthropathy resulting from acute calcium pyrophosphate dihydrate (CPPD) crystal deposits in the joints. Pseudogout can have multiple presentations, ranging from asymptomatic to more severe destructive polyarticular arthritis [1]. Patients may present with fevers and chills lasting weeks, and lab results may be nonspecific for inflammatory or infectious etiologies [2]. Cellulitis is a much more common cause of inflammation caused by a break in the skin barrier resulting in bacterial infection of the skin's subcutaneous and deep dermis layers [3]. Systemic involvement is also present, and patients may have leukocytosis, elevated C-reactive protein levels, and increased erythrocyte sedimentation rate [2]. Due to its non-specific symptomology, cellulitis can mimic many other conditions, including gout, osteoarthritis, septic arthritis, and pseudogout [4]. Despite multiple reports of gout misdiagnosed as pseudo-cellulitis, few studies have compared these diseases [5-8]. This report describes two cases initially misdiagnosed as cellulitis and later identified as CPPD and successfully treated. 

## Case presentation

Case 1

An 83-year-old female presented to the Good Samaritan Hospital (Lebanon, USA) emergency room via emergency medical services on August 8, 2021, with complaints of bilateral leg swelling and pain lasting two days. She described a burning, non-radiating, dull ache, which was worse in the left foot. The pain, rated 8/10 on the pain scale by the patient, was exacerbated by ambulation. Her left foot was extremely tender on palpation. A blister also was present on the left foot.

She had a medical history of hypertension, type 2 diabetes with insulin use, mixed hyperlipidemia, glaucoma, left foot fracture and metal plate insertions, right nephrectomy in 2014 for renal cell carcinoma, anemia of chronic disease, parathyroidectomy for parathyroid adenoma in 2015, and open reduction and internal fixation of right femur in 2020. The patient was also diagnosed with CPPD in April 2019 after discovering intracellular pyrophosphate crystals in a fluid analysis of a left ankle joint aspiration. However, the patient did not follow up with rheumatology, and appropriate treatment was not sought. On admission, she was taking amlodipine, metoprolol, hydralazine, lovastatin, and multiple eye drops. A review of systems was positive for bilateral edema and negative for trauma, fevers, chills, chest pain, and shortness of breath.

**Figure 1:**
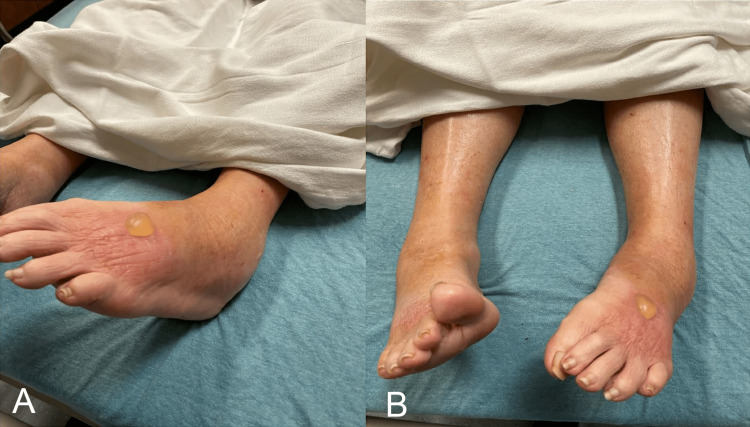
Lower extremity physical findings showing left ankle swelling and a bulla on the dorsal aspect of the left foot

On arrival, the patient was vitally stable with a blood pressure of 134/59 mmHg, heart rate of 89 beats per minute, respiratory rate of 18 breaths per minute, a temperature of 37.7 °C (99.9 °F), and oxygen saturation (SaO2) of 96% on room air. On examination, she appeared uncomfortable with limited lower extremity motion, marked left ankle swelling with warmth and erythema, and a bulla on the dorsal aspect of the left foot (Figure 1 A & B). Her sensation, strength, and distal pulses were intact and equal in the bilateral lower and upper extremities. Given the unclear initial picture and the patient’s complex medical history, a broad list of differential diagnoses including cellulitis, venous insufficiency, myxedema, nephrotic syndrome, deep venous thrombosis of the lower extremity, and pseudogout were considered.

**Figure 2:**
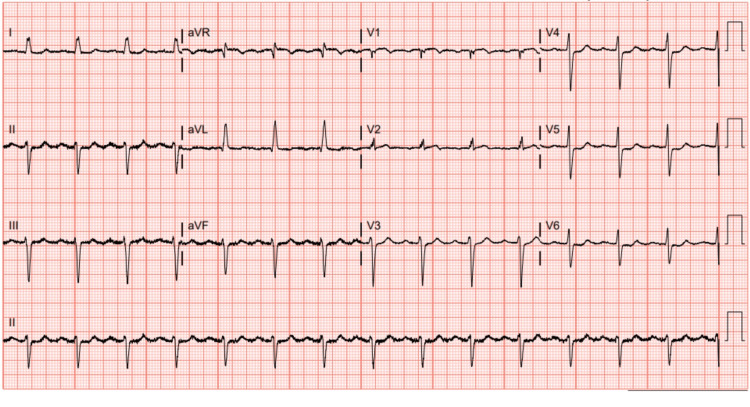
Electrocardiogram in sinus rhythm with left anterior fascicular block and nonspecific ST and T wave abnormalities

**Figure 3:**
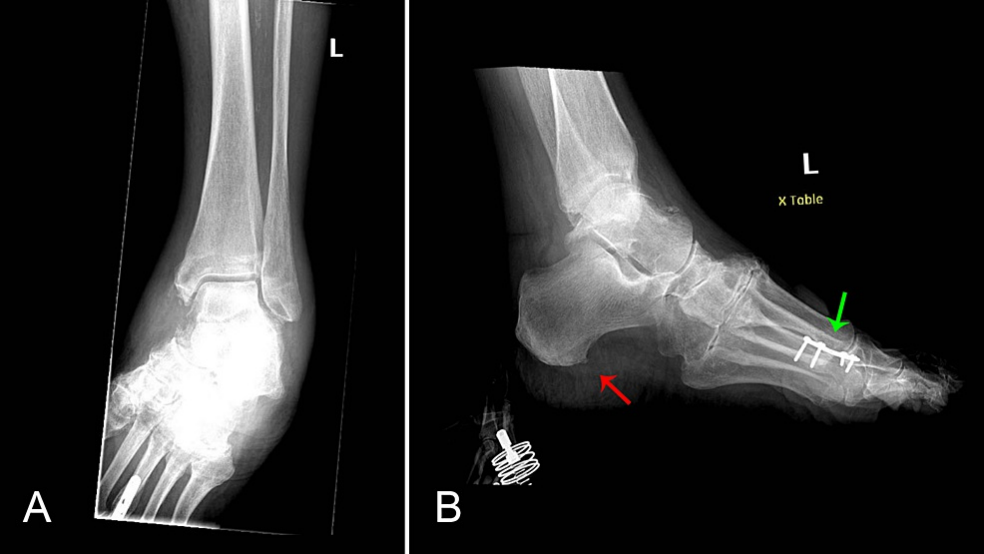
X-rays of the left foot and left ankle show a metallic plate with screws along the distal diaphysis of the third metatarsal bone (green arrow), a small calcaneal spur (red arrow), some osteopenia, degenerative changes in tarsal metatarsal joints, and soft tissue swelling

Initial laboratory results were significant for troponin elevation of 0.04 ng/mL, which increased to 0.11 ng/mL (reference range: <0.03 ng/mL); B-type natriuretic peptide of 277 pg/mL, which increased to 380 pg/mL (reference range: <100 pg/mL), C-reactive protein of 134.9 mg/L (reference range: <10 mg/L); and sedimentation rate of 73 mm/hr (reference range: 0-30 m/hr). An electrocardiogram (EKG) showed a new left anterior fascicular block and new nonspecific ST and T wave abnormalities (Figure 2). A plain-film chest radiograph showed mild cardiomegaly. A bilateral lower extremity ultrasound was unremarkable. The X-rays of the left foot and ankle showed a small calcaneal spur, degenerative changes in tarsal metatarsal joints, and soft tissue swelling (Figure 3 A & B). 

The patient was initially treated with cefepime for cellulitis, but after a thorough chart review and discovery of the previous diagnosis of CPPD in her left ankle, antibiotics were stopped and prednisone and colchicine started instead. The patient found instant relief in symptoms after this medication adjustment. 

Cardiology was consulted due to the elevated troponin and EKG changes, but the patient’s symptoms were not considered true acute coronary syndrome due to the absence of classic symptoms. She was thus treated conservatively for type 1 versus type 2 myocardial infarction (MI) with aspirin, metoprolol, and lovastatin. The patient was discharged on August 24, 2021, to acute rehab with a prednisone taper and continued colchicine for pseudogout prophylaxis. Colchicine was discontinued after discharge from acute rehab. 

Case 2

A 73-year-old male with a medical history significant for atrial fibrillation, cardiomyopathy, chronic obstructive pulmonary disease, previous deep venous thrombosis, hypertension, hyperlipidemia, and stage III adenocarcinoma of the lung was referred to the Good Samaritan Hospital emergency room from the orthopedic clinic on February 20, 2022, for atraumatic left wrist pain and swelling.

The patient first noticed atraumatic left thumb pain two days earlier after receiving chemotherapy through a right upper extremity port. Over the next few days, he noted an increase in pain and swelling in his left hand tracking proximally into his wrist. He rated the pain as 6/10 overall, periodically increasing to 10/10. There was associated warmth and erythema, but he denied trauma to the area, fevers, chills, and any history of inflammatory arthritis or gout. He was taking hydrocodone and acetaminophen in combination on an as-needed basis, providing mild relief for his pain.

During the in-office orthopedics evaluation, potential differential diagnoses for his symptoms were discussed, including trauma, cellulitis, septic arthritis, flexor tenosynovitis, and inflammatory arthritis. The orthopedics team felt he was demonstrating signs of cellulitis in the left hand and would benefit from evaluation in the emergency department, along with required lab work and a medicine consult for possible admission for IV antibiotics. In the emergency department, the patient’s vital signs were within normal limits, and he was afebrile. His physical exam was remarkable only for diffuse swelling of the left wrist and hand without any obvious soft tissue injury. There was warmth but no erythema and acute tenderness of the triangular fibrocartilage complex, scaphotrapeziotrapezoid joint, and thumb extensor retinaculum. Strength was intact in the upper and lower extremities. The sensation of the median, radial and ulnar nerves was intact on the left hand, with easily palpable radial and ulnar pulses.

**Figure 4:**
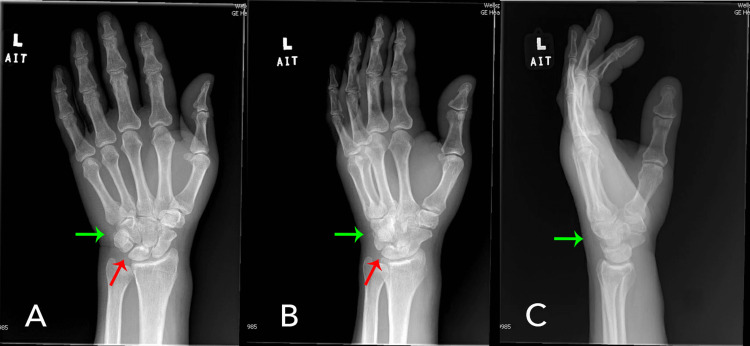
X-rays of the left hand show mild soft tissue calcification of the wrist in the triangular fibrocartilage complex (green arrow) and between the lunate and triquetral bones (red arrow)

**Figure 5:**
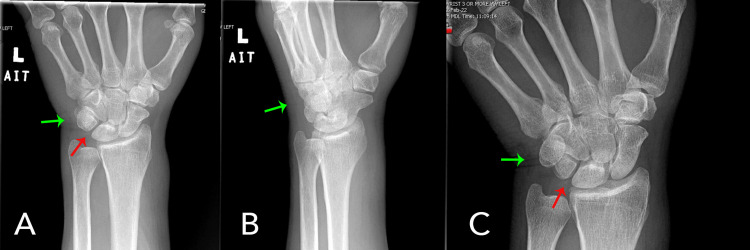
X-rays of left wrist showing mild soft tissue calcification of the wrist in the triangular fibrocartilage complex (Green Arrow) and between the lunate and triquetral bones (Red Arrow)

Initial laboratory results were significant for elevated creatinine from his baseline, indicating a possible acute kidney injury. Additionally, he had low hemoglobin and platelets but was at his baseline given his cancer history. His C-reactive protein was 38.3 mg/L (reference range: <10 mg/L) and the sedimentation rate was 34 mm/hr (reference range: 0-30 mm/hr), representing an inflammatory process. The X-rays of the left hand (Figure 4 A & B) and wrist (Figure 5 A & B) showed mild soft tissue calcification of the wrist in the triangular fibrocartilage complex and between the lunate and triquetral bones.

Sepsis was considered as tachycardia may be masked by the beta-blocker, and fever may be masked by acetaminophen. His white blood cell count also may have been falsely normal due to his immunocompromised state, but clinically the patient did not appear septic. We initiated intravenous fluids and antibiotics for 24 hours for possible cellulitis and stopped when blood cultures came back negative. 

Due to localized tenderness of the joints, pseudogout was suspected. A loading dose of colchicine and 40 mg of prednisone were initiated. The next day, the swelling had subsided. The patient was instructed to continue taking 40 mg prednisone for three more days to complete a total of five days and to take 0.6 mg colchicine twice daily for the next 14 days, and then take 0.6 mg once daily until seen by rheumatology. 

## Discussion

We describe two cases of CPPD initially suspected to be other conditions. Upon diagnosis, both patients’ symptoms were relieved. These two cases highlight how CPPD can be misdiagnosed because its symptomology mimics other more common diagnoses for acute inflammation, such as cellulitis. Cellulitis has an estimated prevalence of 14 million cases annually [9]. Despite its prevalence, there is no established gold standard or reference for diagnosis. Moreover, symptoms of cellulitis are often nonspecific and observed in several non-infectious conditions, including CPPD, which can have multiple presentations and lab results that are nonspecific for inflammatory or infectious etiologies [1]. Thus far, attempts to establish a definitive diagnosis lack specificity, feasibility, or sufficient evidence to be used in a healthcare setting [10]. 

Misdiagnosis is a particular concern in the geriatric population due to the significant risk of morbidity. In the United States, about 30% of cellulitis diagnoses in the emergency department are misdiagnosed, contributing to antibiotic resistance and unnecessary healthcare costs [10]. A retrospective cross-sectional study by Weng et al. looked at the consequences of misdiagnosing lower-extremity cellulitis and found that 79 of 269 (30%) emergency department patients were incorrectly managed; of those, 85% did not require any hospitalization and 92% received unneeded antibiotic treatment [11]. The study estimates that more than 44,000 patients with pseudo-cellulitis are exposed to unnecessary antibiotics annually in the United States, leading to readmission rates of up to 13% and multiple complications such as GI distress, anaphylaxis, nosocomial infections, and antibiotic resistance [11]. 

Cellulitis frequently affects older adults, with incidence increasing by 43% with every additional decade of age [3]. Geriatric patients who are correctly diagnosed with pseudo-cellulitis are 86% less likely to experience complications of treatment [12]. In a study by Bailey et al., 17% of patients with suspected cellulitis were found to have other conditions, most commonly stasis dermatitis, gout, pseudogout, and hematoma [13]. Moreover, 8.9% of the pseudo-cellulitis cases were found to have gout or pseudogout. Warmth at the site may be a good predictor for true cellulitis, with a 2.2-fold increase in likelihood, whereas erythema, tenderness, and edema are not seen to be good predictors [12]. Predisposing risk factors (e.g., diabetes, malignancy, and IV drug use) also show a 90% increased likelihood of true cellulitis [13]. 

Patients with CPPD may have negative x-rays, and about 40% of patients have no radiological findings [14]. Nevertheless, positive birefringent rhomboid-shaped crystals in the affected joint’s synovial fluid indicate a definitive diagnosis of pseudogout. Pseudogout also may be suspected in patients with localized inflammation around one or more joints with a severely limited range of motion. The distribution of joint involvement and prior history of pseudogout can assist in forming a diagnosis [10]. A dermatological consult also may be useful in cases with an unclear diagnosis. A clinical trial by Arakaki et al. found that outpatient dermatological consultations can help rule out cellulitis and thus reduce unnecessary antibiotic use [4]. 

Further studies should be conducted to determine how to definitively diagnose cellulitis to reduce confusion between the disease and other causes of inflammation. Improving these diagnostic criteria will allow for appropriate integration of antibiotic stewardship, reduce hospital costs, and prevent hospital-acquired complications. 

## Conclusions

Gout and pseudogout represent challenging diagnoses, particularly in elderly populations due to atypical presentations and underlying comorbidities. It is therefore important to consider pseudo-cellulitis presentations such as CPPD as possible diagnoses for any patient presenting with cardinal signs of inflammation in any joint. Early and accurate diagnoses help physicians provide adequate and timely patient care to avoid unnecessary hospitalization and antibiotic use. It is essential that healthcare providers understand the underlying pathophysiology of CPPD to differentiate it from other forms of inflammatory arthritis and soft tissue inflammation. 
